# Microfluidic Electrochemical Impedance Spectroscopy of Carbon Composite Nanofluids

**DOI:** 10.1038/s41598-017-00760-1

**Published:** 2017-04-07

**Authors:** Hye Jung Lee, Seoung-Jai Bai, Young Seok Song

**Affiliations:** 1 Department of Fiber System Engineering, Dankook University, 126 Jukjeon-dong, Suji-gu, Yongin-si, Gyeonggi-do 448-701 Korea; 2 Department of Mechanical Engineering, Dankook University, 126 Jukjeon-dong, Suji-gu, Yongin-si, Gyeonggi-do 448-701 Korea

## Abstract

Understanding the internal structure of composite nanofluids is critical for controlling their properties and engineering advanced composite nanofluid systems for various applications. This goal can be made possible by precise analysis with the help of a systematic robust platform. Here, we demonstrate a microfluidic device that can control the orientation of carbon nanomaterials in a suspension by applying external fields and subsequently examine the electrochemical properties of the fluids at microscale. Composite ﻿nanofluids were prepared using carbon nanomaterials, and their rheological, thermal, electrical, and morphological characteristics were examined. The analysis revealed that microfluidic electrochemical impedance spectroscopy (EIS) in the device offered more reliable in-depth information regarding the change in the microstructure of carbon composite nanofluids than typical bulk measurements. Equivalent circuit modelling was performed based on the EIS results. Furthermore, the hydrodynamics and electrostatics of the microfluidic platform were numerically investigated. We anticipate that this microfluidic approach can serve as a new strategy for designing and analyzing composite nanofluids more efficiently.

## Introduction

Composite nanofluid, a relatively new class of liquid suspension with nanosized composite particles (<100 nm) in at least one dimension, exhibits fascinating potential with exceptionally enhanced physical features, such as thermal, electrical, and rheological properties compared with the properties of the base fluid^1–3^. Basically, composite nanofluids require a stable dispersion of embedded composite nanoparticles and low hydrodynamic resistance for various applications such as energy, automobiles, welding equipment, laser diode arrays, microwave tubes, and so on^4–8^. In many cases, those applications are implemented using microstructural channels through which nanofluids flow in order to enhance the corresponding heat transfer efficiency^9, 10^. It is desirable to develop a measurement platform that can perform microscopic analysis but not macroscopic analysis in an effort to generate more precise and refined data with a few samples^11^. The use of microfluidic devices can allow one to look into the physicochemical properties of material systems in a controlled manner with the help of optical, electrical, and electrochemical techniques^12–14^. In particular, microelectrode-embedded microfluidic devices have the capability to provide more integrated and efficient platforms due to their ease of fabrication.

Carbon nanomaterials have been extensively studied and are regarded as fascinating next generation matter for a wide range of applications including nanotechology, biotechnology, energy, and environment^15–19^. For instance, graphene shows exceptionally high physicochemical properties for electrochemical applications, such as energy storage, energy transport, electrochemical sensor and actuator, and electrocatalysis, owing to its unique two dimensional structure^20, 21^. Much research has, however, been done into the solid state based properties of carbon materials^22^. While composite nanofluids with carbon nanomaterials have also been investigated in many fields, few studies on the microstructure of composite nanofluids have been reported^3^. Indeed, it is critical to control the structure and morphology of composite nanofluids in a systematic fashion and to take advantage of their unique properties. On the other hand, the advent of carbon nanomaterials such as carbon nanotubes and graphenes might open a path for designing and fabricating an engineering material with maximized properties.

Carbon nanomaterials in a suspension are oriented along the electric and magnetic fields applied externally, thus leading to significant enhancement of the electrochemical and physical performance of the suspension^23–26^. Graphitic carbon materials have different electrochemical behaviors depending on the number of single layer graphene and the structural features of the material^22, 27^. The graphene edge has much higher specific capacitance but lower electrical and thermal conductivities than the graphene basal plane^28^. In this sense, controlling the orientation of carbon nanomaterials is important to engineer material systems in a more active manner^29–34^.

In the current study, we develop a microfluidic platform that can apply external stimuli and simultaneously analyze the electrochemical properties of a composite suspension at microscale. The platform enables us to fine tune the material structure, intensify the external fields in the system, and precisely detect the resulting response with a small volume. Carbon composite nanofluids were prepared by embedding carbon nanomaterials in the suspension and their physicochemical properties were analyzed. The orientation and microstructure of the carbon nanomaterials in the fluids were microscopically controlled with the help of an electric field and a shear field and the resulting change in the electrochemical impedance was examined.

## Results and Discussion

**Figure 1:**
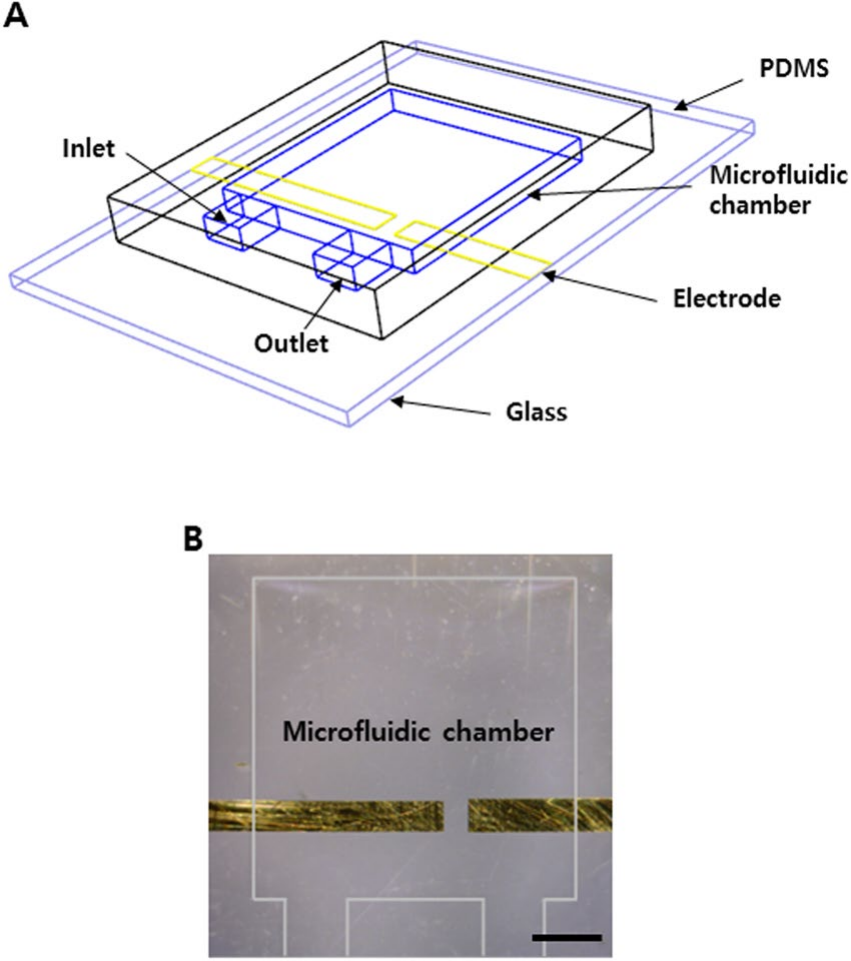
(**A**) 3-D schematic illustration of the microfluidic device and (**B**) top-view image of the device with the embedded gold electrodes.

Figure 1(A) presents the entire design of the microfluidic device fabricated in this study. The device was designed to examine the effect of the applied electrical and flow fields on the internal structure of carbon composite nanofluids. It contains a microfluidic chamber, an inlet and an outlet. On the other hand, the microfluidic chamber has a sufficiently large area to ensure a relatively stable internal structure of the carbon composite nanofluids over the two electrodes embedded in the device for electrochemical measurements. The fabricated microfluidic device is depicted in Fig. 1(B). The patterned electrodes were soldered for the connection with a measurement device.

**Figure 2:**
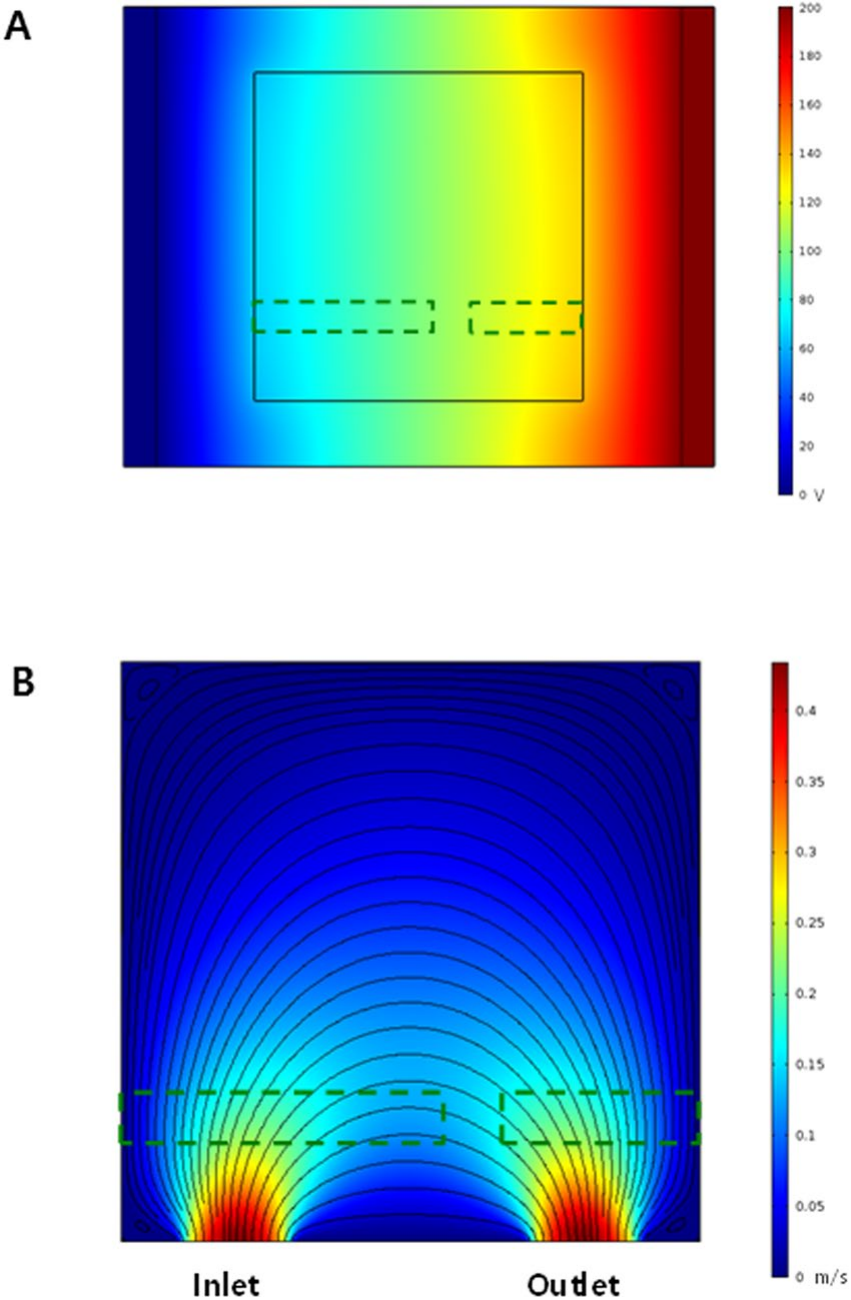
Numerical analyses for the microfluidic device: (**A**) electric field distribution and (**B**) flow field distribution. The dashed lines indicate the positions of the gold electrodes.

The orientation behavior of the carbon nanomaterials in the microfluidic devices under external fields including penitential and flow fields can be estimated with the help of numerical simulation. Figure 2 demonstrates the electric field distribution and the flow field distribution in the microfluidic device modelled numerically. It was found that the potential distribution and stream lines were generated across the two electrodes.

**Figure 3:**
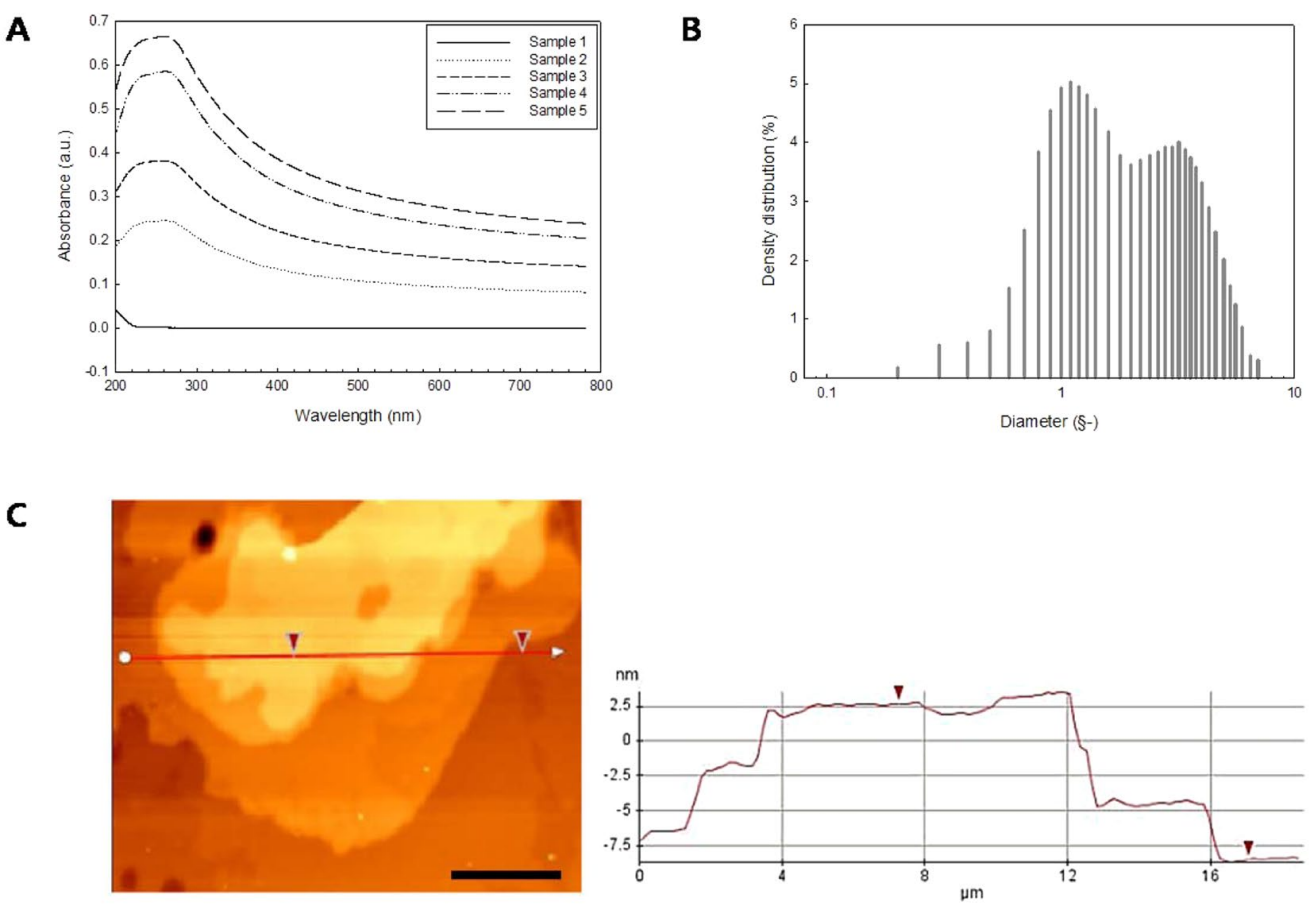
(**A**) UV-visible absorption spectra of the samples, (**B**) size distribution of sample 2, (**C**) AFM results of dispersed graphite. The scale bar indicates 5 μm.

The composition of the samples used in this study is listed in Table S1. The concentrations of graphite and CNT in the suspensions were obtained using the absorbance results presented in Fig. 3(A). For the measurement, the suspensions were diluted one thousand times. The size distribution for sample 2 is demonstrated in Fig. 3(B). The average diameter of graphite measured using a particle size analyzer was around 1 μm, and the average length of CNT was 3 μm. Figure 3(C) shows the results of the AFM measurement. The thickness of graphite was found to be around 10 nm, which corresponds to the thickness of several layers of graphene^35^.

**Figure 4:**
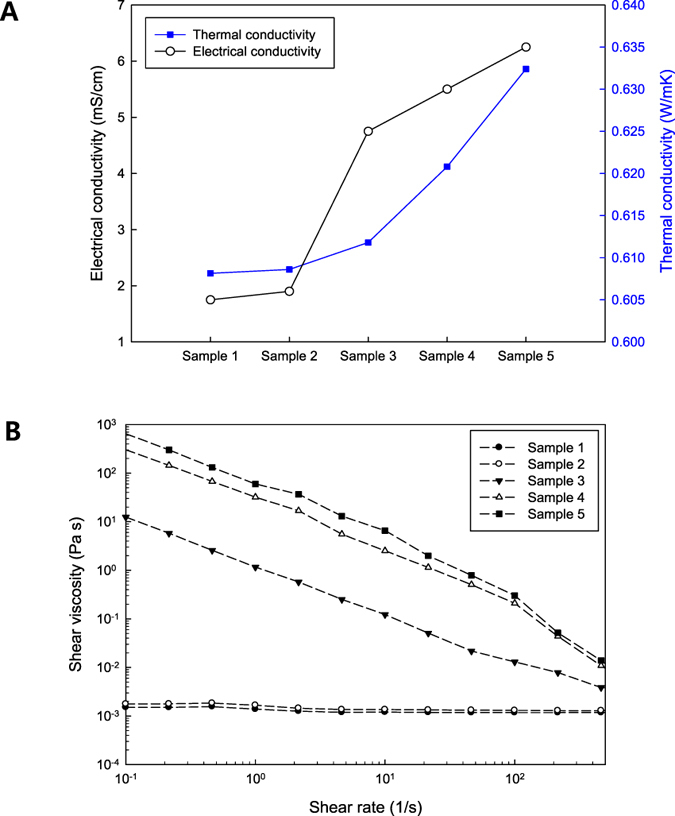
(**A**) Variation in the electrical and thermal conductivities for the samples and (**B**) shear viscosity of the samples as a function of the shear rate.

**Figure 5:**
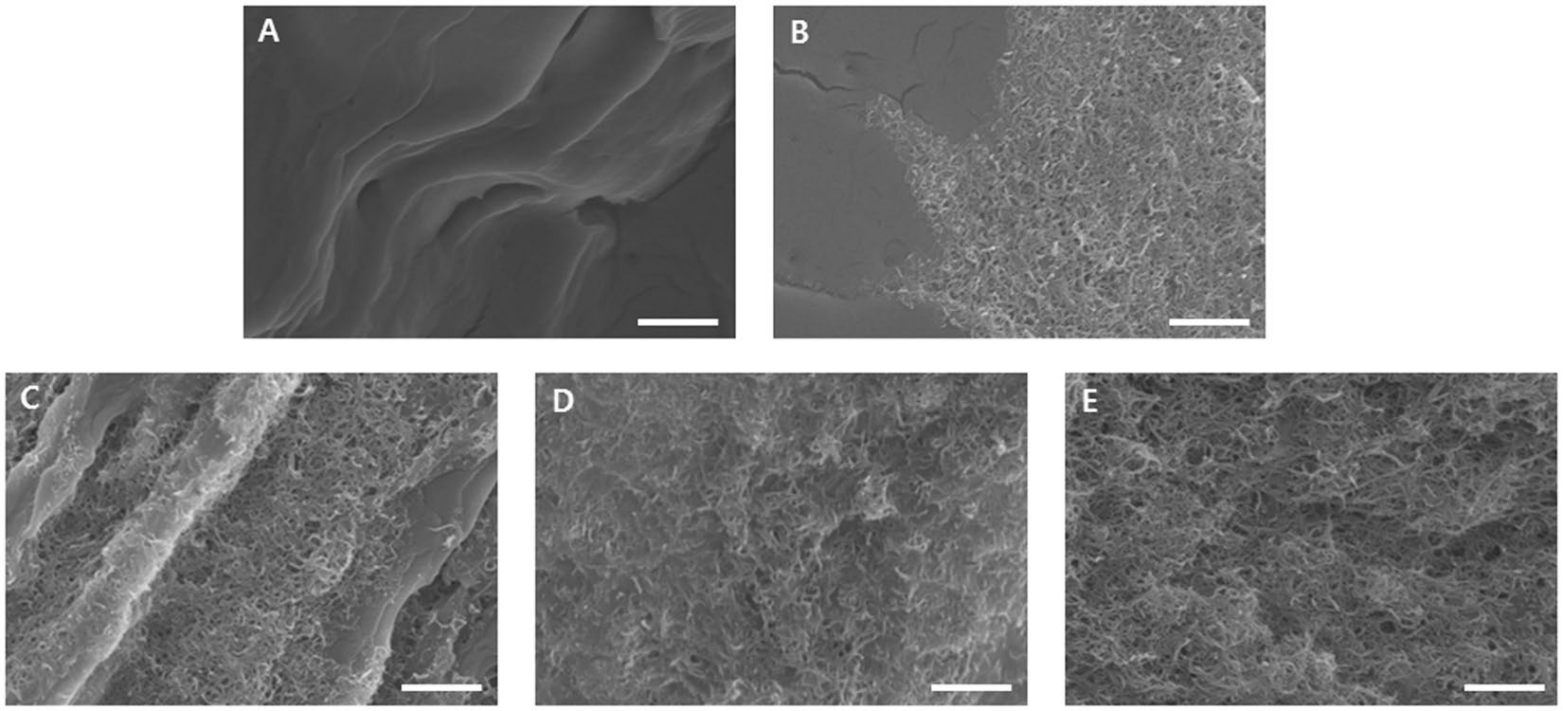
FESEM images of (**A**) sample 1, (**B**) sample 2, (**C**) sample 3, (**D**) sample 4, and (**E**) sample 5. The scale bar indicates 1 μm.

Figure 4(A) demonstrates the electrical and thermal conductivities of the composite specimens prepared. The electrical conductivities were found to increase as the content of CNTs increased. In particular, there was a relatively large enhancement between sample 2 and sample 3. Interestingly, this jump in electrical conductivity was accompanied by a four order of magnitude increase in the shear viscosity as shown in Fig. 4(B). The embedded CNTs yield the improvement in the electrical and thermal conductivities of the suspensions compared with those of the base fluid. For the thermal conductivities, the particle concentration, size, dispersion and stability can act as an important factor for the increment. To understand the thermal characteristics of the composite nanofluids, analytic evaluation was carried out using the Hamilton-Crosser model, which has been employed for the prediction of the thermal conductivity of suspensions as explained in Supporting Information^18^. It was found that the thermal conductivities obtained experimentally were higher than the analytic values (i.e., 1~2% ratio enhancement with respect to the liquid value according to the particle concentration). This discrepancy might be explained with two effects: one is the presence of organized structures such as percolation structures in the carbon nanomaterials, and the other is the contribution of the Brownian motion of the particles. The rheological characteristics of the nanofluids as a function of the shear rate are presented in Fig. 4(B). As the shear rate increased, the shear viscosities decreased. This is the so-called shear thinning or pseudoplastic phenomenon. When a shear stress is imposed to carbon composite nanofluids, the CNTs are aligned in the shear flow direction. As a result, the hydrodynamic flow resistance diminishes. The higher content of CNTs led to the stronger shear thinning behavior and higher viscosities of the nanofluids. In particular, the viscosities at the low shear rate region were significantly affected by the weight fraction of the CNTs. To analyze the rheological behavior quantitatively, we adopted a popular generalized Newtonian fluid model, the power law model ($\tau =K{\dot{\gamma }}^{n})$. Here, *τ* is the shear stress, $\dot{\gamma }$ is the shear rate, and *K* is the flow consistency index (Pa s^n^). The calculated power law index *n* was presented in Table S2. As mentioned previously, there exists a drastic increase in the shear viscosity, which indicates the presence of a rheological percolation structure between 0.056 wt% and 0.106 wt% of the CNT concentration. Furthermore, the addition of CNTs plays a more critical role in generating the rheological network structure than the addition of graphite. The FESEM images of the specimens are shown in Fig. 5. As the amount of CNTs was increased, the entanglement of CNTs was intensified.

**Figure 6:**
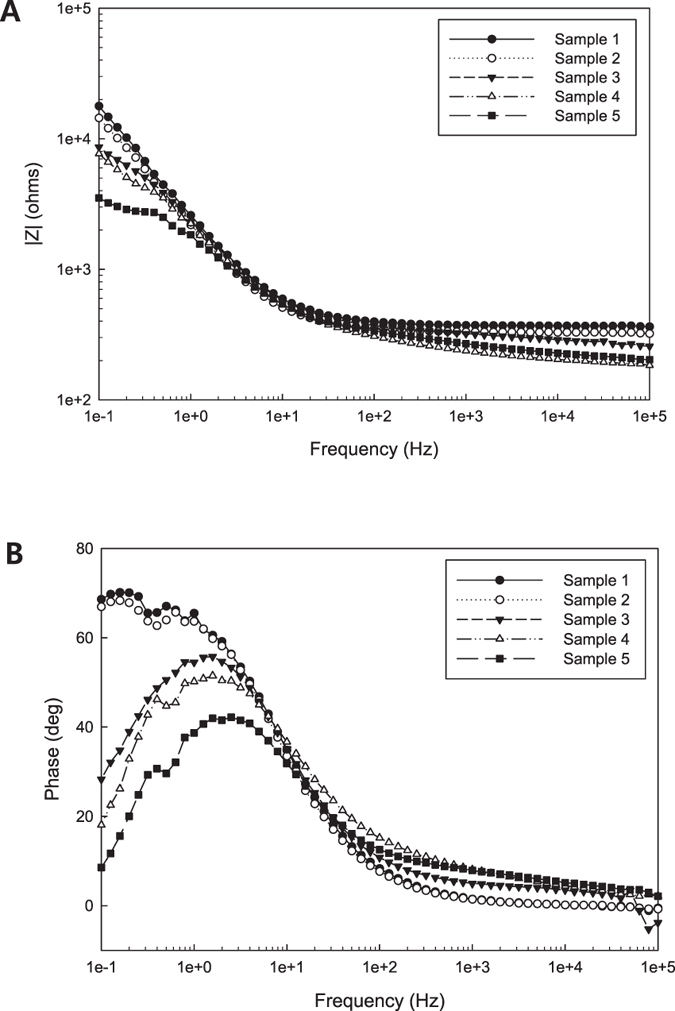
Impedance results of the bulk solution: (**A**) magnitude and (**B**) phase of the Bode plot.

Figure 6(A) and (B) show the impedance change in the composite nanofluids as a function of frequency. The magnitude of impedance measured for the bulk solution at macroscale decreased with an increase in the concentration of the CNTs. In particular, samples 3, 4 and 5, with the larger CNT content, possessed the low impedances notably in both high and low frequency regimes. The ohmic behavior of samples 3, 4 and 5 in the low frequency regime implies that the CNTs are perfectly entangled with each other. Figure 6(B) displays the phase of the Bode plots for the samples.

Equivalent circuit modelling was carried out to evaluate the change in the internal structure of the composite suspensions at macroscale. Table 1 presents an equivalent circuit containing simple electrical components, *R*_*CT*_, CPE, *C*_*DL*_, and *R*_*S*_. These indicate the charge transfer resistance, the constant phase element, the double layer capacitance for the working electrode, and the resistance for the composite nanofluid, respectively. The selection of the components was made considering the electrical path in the measurement system. In order to take into account the Warburg impedance, a constant phase element was used:

1 $${Z}_{CPE}=\frac{1}{C{(i\omega )}^{P}}$$

where C is the capacitance, and *ω* is the angular frequency. The CPE acts as the Warburg impedance when P becomes 0.5. In the current study, the main interesting component of the equivalent circuit is the resistance for the conductive path generated by the carbon nanomaterials. The electrochemical impedance spectroscopy (EIS) results for samples 2 and 3 were fitted, as listed in Table 1. The results revealed that sample 3, with a higher content of CNTs, had a lower suspension resistance than sample 2. This is consistent with the electrical conductivity results presented in Fig. 4(A).

**Table 1:** Summary of the values of electrochemical components used for fitting the results of bulk measurement.

Equivalent circuit	Calculated impedance data		Sample 2	Sample 3
	Working electrode	R_CT_ (Ω)	43.98	58.17
		CPE_E_–C (F)	9.764 × 10^−5^	1.216 × 10^−4^
		CPE_E_–P	0.7698	0.7361
		C_DL_ (F)	1.699 × 10^−5^	8.024 × 10^−7^
	Nanofluid	R_S_ (Ω)	329.2	274.9

**Figure 7:**
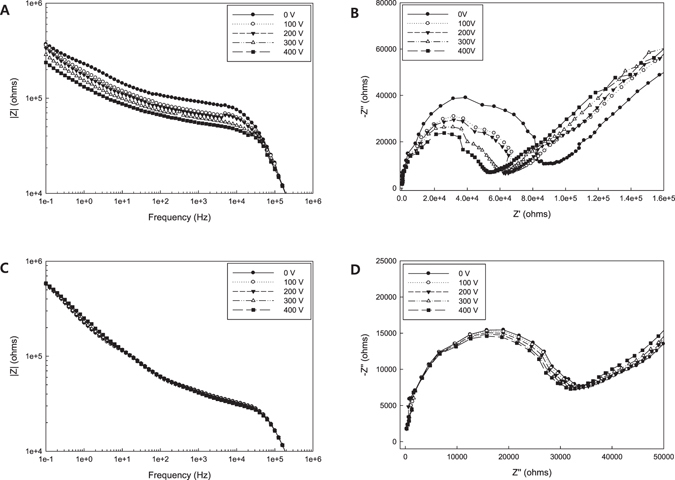
Microfluidic impedance changes according to the applied potentials: (**A**) Bode plot of sample 2, (**B**) Nyquist plot of sample 2, (**C**) Bode plot of sample 3, and (**D**) Nyquist plot of sample 3.

Figure 7 illustrates the impedance changes measured in the microfluidic device when applying electrical potentials. It has been reported that carbon nanomaterials such as graphene and carbon nanotubes are aligned in the direction of the applied external electrical field. Basically, macroscopic measurement to determine the electrochemical features of composite nanofluids is not appropriate for accurate observation of microstructural changes due to the large dimensional differences and low uniformity of materials. In this sense, the microfluidic measurement proposed in this paper can allow one to probe the composite nanofluids with intensified material properties at microscale. As the external electric potential was increased, the impedance of the nanofluids decreased (Fig. 7(A)). This is attributed to the orientation of the carbon nanomaterials dispersed in the nanofluids as a result of the electric field and the corresponding anisotropy of the electrical properties for the nanomaterials. Furthermore, we need to consider that the dangling bond of graphitic materials can act as a reactive site with superior electron transfer characteristics to the graphitic C-C bond in the basal plane. Figure 7(B) shows the Nyquist plot for sample 2. The radius of the hemispheres indicating the contribution of the charge transfer resistance *R*_*CT*_ decreased with increasing potential. On the other hand, the effect of the electric field was not significant in the results for sample 3 as shown in Fig. 7(C) and (D). This result can be explained in two aspects. First, the edge orientation of the suspended materials can facilitate charge transfer from the nanofluid to the electrode. It is, however, assumed that since sample 3 has a sufficiently high CNT content for generating the network structure of CNTs, the orientation of the nanomaterials induced by the potential cannot play a key role in the impedance behavior unlike in sample 2. Second, the higher concentration of entangled CNTs in the sample 3 prevented them from being aligned under the external electric field. Therefore, the change of the impedances was not evident as the external electric field was intensified.

**Figure 8:**
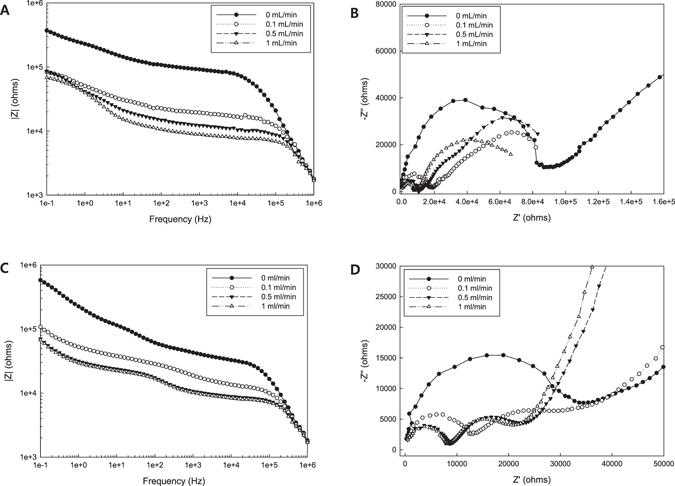
Microfluidic impedance changes according to the applied flow fields: (**A**) Bode plot of sample 2, (**B**) Nyquist plot of sample 2, (**C**) Bode plot of sample 3, and (**D**) Nyquist plot of sample 3.

**Figure 9:**
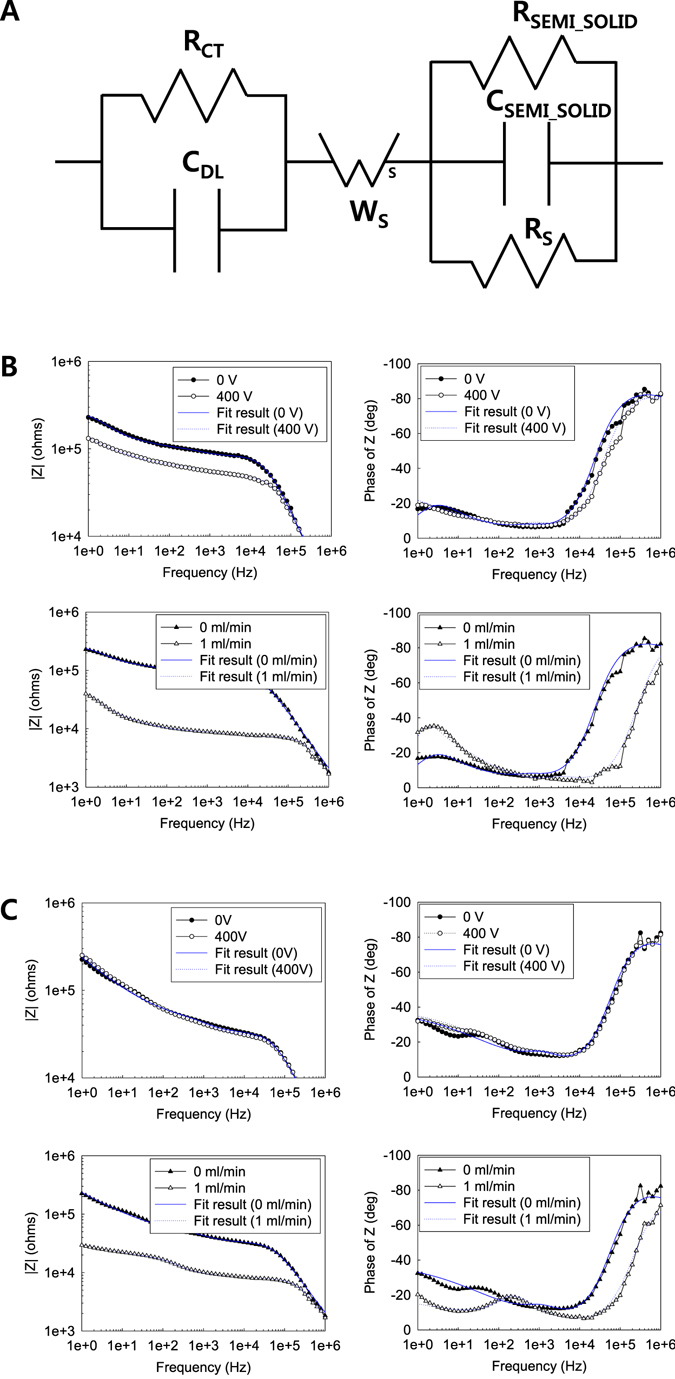
Equivalent circuit analysis: (**A**) equivalent circuit for microfluidic impedance fitting and (**B**) fitting results of sample 2 and (**C**) fitting results of sample 3.

In order to manipulate the orientation of carbon nanomaterials, hydrodynamics was applied to the microfluidic device in this study. Figure 8(A) and (B) show the Bode plot and the Nyquist plot for the sample according to the imposed flow field, respectively. Similar to the response to the electric potential, the shear stress in microfluidic devices causes particles to align along the stream lines in the channel. Consequently, the higher velocity of flow leads to lower impedance for the composite nanofluids in the channel. Indeed, the electrochemical impedance spectroscopy (EIS) helps provide in-depth understanding of the microstructural change in response to external stimuli. When imposing a velocity field of 0.1 ml/min to the device, a drastic drop in the impedance was developed. Basically, the electrochemical impedance spectroscopic feature is determined by two different mechanisms: one is the electron transfer behavior of the electrode, and the other is the mass-transfer behavior of electrochemical elements. Unlike the impedance results of the composite nanofluids under the electrical potential, sample 3 presents the dependency of the impedance on the flow field applied to the device (Fig. 8(C) and (D)). In particular, the cole-cole plot shows the complicated behavior of the composite nanofluids according to the flow field. The applied flow field can lead to not only the orientation of carbon nanomaterials but also the increased convection of electrochemical elements towards the electrode, thereby resulting in complex flow field dependency for the electrochemical properties. Compared to the macroscopic analysis of impedance, the microfluidic EIS platform allows us to explore a complex material system at microscale. Therefore, a different equivalent circuit needs to be adopted for evaluating the microfluidic EIS as illustrated in Fig. 9(A). The image shows additional components such as *R*_*SEMI_SOLID*_ and *C*_*SEMI_SOLID*_, reflecting the resistance and the capacitance of the suspended nanomaterials in the nanofluid, respectively. Additionally, since the microscale environment of the channel limits the mass transfer of the electrochemical elements, a finite Warburg impedance which was denoted as Ws in the equivalent circuit was employed instead of the CPE in the macroscale analysis. The relevant fitting results presented in Fig. 9(B) and (C) reveal that the equivalent circuit data are in accordance with the experimental results. The values for the electrochemical components used for fitting the results of the microfluidic impedance measurements are listed in Table 2. It is interesting to note that the *R*_*SEMI_SOLID*_ diminished under the electric potential and the flow field compared with the values of the control sample. In this way, the microfluidic EIS introduced in the present study was successfully employed as a novel tool for analyzing composite nanofluids in combination of microfluidic and electrochemical systems.

**Table 2:** Summary of the values of electrochemical components used for fitting the results of microfluidic impedance measurements.

Calculated impedance data		Sample 2			Sample 3		
		Control	400 V	1 ml/min	Control	400 V	1 ml/min
Working electrode	R_CT_ (Ω)	79814	46128	7274	29746	27844	7017
	C_DL_ (F)	8.562 × 10^−11^	8.825 × 10^−11^	8.535E × 10^−11^	9.502 × 10^−11^	9.304 × 10^−11^	1.033 × 10^−11^
	W_S_-R	182390	448940	45596	5.84E + 6	6.6039E + 6	78756
	W_S_-T	0.23853	10.71	0.36733	381.3	310.2	29.66
	W_S_-P	0.43648	0.39573	0.52029	0.43017	0.4414	0.27876
Nanofluid	R_SEMI__S_OLID_ (Ω)	5.777 × 10^9^	9.201210^7^	6.12310^6^	78630	37724	21237
	C_SEMI_SOLID_ (F)	2.7213 × 10^−8^	2.4672 × 10^−8^	4.4893 × 10^−8^	2.2457E × 10^−8^	2.1775 × 10^−8^	1.6588 × 10^−7^
	R_S_ (R)	10738	8853	1026	8330	8073	8019

## Conclusions

We developed a microfluidic device that can control the orientation of carbon nanomaterials and analyze the electrochemical properties of composite nanofluids from a microscopic perspective. Composite nanofluids were prepared by dispersing carbon nanomaterials, and their physical properties including rheological, thermal, electrical, and morphological characteristics were evaluated. The electrochemical impedance spectroscopy (EIS) analysis for the carbon composite nanofluids was carried out at two different scales, i.e., in macroscopic and microscopic environments. On the basis of the EIS results, the corresponding equivalent circuits were proposed. Electric fields and flow fields were imposed to the microfluidic platform in a bid to control the microstructure of the carbon composite nanofluids. Numerical simulations of the hydrodynamics and electrostatics for the microfluidic platform were conducted to analyze the effect of the external fields on the orientation of the carbon nanomaterials in the device. The findings show that microfluidic EIS provides robust and reliable results for the change in the internal structure of composite nanofluids compared with typical bulk measurement. We foresee that the microfluidic approach proposed in this study is poised to serve as a new strategic pathway for designing and analyzing carbon composite nanofluidic systems at the interface of material engineering and MEMS technology.

## Experimental

### Preparation of ﻿composite nanofluids

Synthetic graphite was purchased from Sigma Aldrich, USA, with a powder size lower than 20 μm. Multiwall carbon nanotube (MWCNT) with a diameter of 10~15 nm and a length of 10~20 μm was supplied by Iljin Nanotech, Korea. Sodium dodecyl sulfate (SDS) was used as a surfactant to disperse graphite and MWCNT uniformly and stably. SDS of 2.0 wt% was prepared with distilled water, and the graphite and MWNCT were dispersed in the solution. The mixture was sonicated for 1 h and kept in a stationary state for 12 h before removing sediments of the particles. After that, only the supernatant (top 20%) from the suspension was used for experiments.

### Design of microfluidic chamber

A microfluidic device was designed and fabricated to analyze the electrochemical impedance spectroscopy of carbon composite nanofluids. The microfluidic chamber had a dimension of 5000 × 5000 × 12 μm^3^, which is large enough to develop uniform hydrodynamic or electric fields. For electrochemical measurements, two electrodes were placed on a glass slide. The width of the electrodes was 500 μm.

### Fabrication of a microfluidic device

The microfluidic chamber was prepared by bonding a polydimethylsiloxane (PDMS) device with a glass substrate containing the patterned gold electrodes. First, an SU8 mold on a silicon wafer was prepared using photolithography. A fluorocarbon solution was coated on the mold surface to enhance the hydrophobicity of the mold surface, thus increasing the ease of peeling the PDMS replica. A PDMS pre-polymer (Sylgard 184, Dow Corning, USA) and a curing agent were mixed at a 10:1 (w/w) ratio and poured onto the SU8 mold wafer. Thereafter, the PDMS mixture was cured at 70 °C for 3 h, and the replica was detached from the mold wafer. Inlet and outlet holes were generated by punching the PDMS replica. For the electrode fabrication, Cr/Au films for the electrodes were sputtered on a glass substrate with a thickness of 50/500 nm using shadow masks. The PDMS replica and the glass substrate with the two electrodes were exposed to O_2_ plasma for bonding. After the treatment, the microfluidic chamber of the PDMS replica and the electrodes on the glass substrate were aligned and pressed for permanent bonding.

### Measurements

The transmittance of solution was measured using UV-VIS spectroscopy (Lambda 25, PerkinElmer) to obtain the concentration of particles in the suspension. X-ray diffraction (XRD) experiments were carried out using a Bruker D8-Advance X-ray diffractometer with Cu Kα radiation (λ = 1.5406 Å). The particle size distribution was measured with a particle size analyzer (Cilas 1090). To analyze the morphology of the samples, the composite suspensions were freeze-dried, and imaged with a field emission scanning electron microscopy (FE-SEM, S-4300, Vecco) at an acceleration voltage of 5 kV. The carbon nanoparticles were analyzed using atomic force microscopy (AFM, Nanowizard I, JPK Instrument). The resonant frequency and spring constant of the cantilever were 300 kHz and 37 N/m, respectively.

The rheological characteristics of the composite suspensions were analyzed by using a rheometer (ARES-G2, TA Instruments) with a parallel plate fixture. For the measurement of the steady shear viscosity, the shear rate applied was from 0.01 to 2000 *s* ^−1^ at a constant shear stress of 1 Pa^36^. The electrical conductivities and thermal conductivities of the composite nanofluids were measured using an electrical conductivity meter (SevenGo Duo, METTLER TOLEDO) and a modified transient plane source technique (MTPS), respectively.

The EIS experiments were conducted at two different scales (i.e., macro- and micro-scales) using a potentiostat, VeraStat 3 (Princeton Applied Research, USA), in a two-electrode system. For the bulk suspension, two Pt wires were employed in the frequency range of 0.1 to 100 kHz. For the microfluidic EIS measurement, two Au micropatterns were used as the working electrode and the counter/reference electrode. The working electrode was polarized at 0 V with respect to the counter/reference electrode while a sinusoidal voltage with an amplitude of 10 mV in the frequencies ranging from 0.1 Hz to 100 kHz was employed. Different electric fields were applied along the electrodes by imposing a potential to two ITO glasses, which were positioned at the side walls of the PDMS. Continuous flow of the composite nanofluids was achieved using a syringe pump.

### Equivalent circuit modeling

An equivalent circuit with simple electrical components was assumed to evaluate the change in the internal structure of the carbon composite nanofluids. A complex nonlinear least-squares (CNLS) method was used for the equivalent circuit design, relevant data processing, and impedance estimation^37^. The data fitting with the equivalent circuit was carried out in a frequency range of 1~100 kHz.

### Simulation

To analyze the external field induced orientation of particles in the nanofluids in the microfluidic device in a more quantitative manner, we carried out numerical simulations of the hydrodynamics and electrostatics for the device. For the modeling of the flow field in the microfluidic chamber, we assumed incompressible and Newtonian flow. The corresponding governing equations are as follows:

2 $$\rho \frac{D{\boldsymbol{u}}}{Dt}=\mu {{\nabla }}^{2}{\boldsymbol{u}}-{\nabla }P+\rho {\boldsymbol{g}}$$

3 $${\nabla }\cdot {\boldsymbol{u}}=0$$

where *μ* is the viscosity of liquid, **u** is the velocity, *ρ* is the density, and *P* is the pressure. When applying a voltage difference between the two ITO electrodes, an electric field was generated across the electrodes. The electric field was calculated using the following equations:

4 $${\boldsymbol{E}}=-{\nabla }V$$

5 $${\nabla }\cdot {\boldsymbol{D}}={\rho }_{v}$$

where ***E*** indicates the electric field, *V* is the electric potential, ***D*** is the dielectric displacement, and *ρ*_*v*_ is the external charge. The parameter values used were taken from the related literature, and a finite element method was used to solve the equations^38^.

## Electronic supplementary material

Supporting Information
