# Supplementary material for: Microfluidic Electrochemical Impedance Spectroscopy of Carbon Composite Nanofluids
Source: Sci Rep. 2017 Apr 7;7:722. doi: 10.1038/s41598-017-00760-1 (PMC5429664; doi:10.1038/s41598-017-00760-1)
Supplement: Supplementary file 1 — Supporting Information [file 41598_2017_760_MOESM1_ESM.pdf]

Supplementary Information:

Microfluidic Electrochemical Impedance Spectroscopy of Carbon Nanofluids

Hye Jung Lee[1#], Seoung Jai Bai[2#], Young Seok Song[1*]

[1]Department of Fiber System Engineering, Dankook University,

126 Jukjeon-dong, Suji-gu, Yongin-si, Gyeonggi-do 448-701, Korea

[2]Department of Mechanical Engineering, Dankook University,

126 Jukjeon-dong, Suji-gu, Yongin-si, Gyeonggi-do 448-701, Korea

*Corresponding author: Young Seok Song

Tel.: +82-31-8005-3567; Fax: +82-31-8005-2209; E-mail: ysong@dankook.ac.kr

[#]These authors contributed equally to this work.

The supplementary contains more information on:

1. **Compositional analysis**
2. **Structural analysis**
3. **Size analysis**
4. **Thermal conductivity**
5. **Rheological analysis**
6. **Morphological analysis**
7. **Electrochemical analysis**

1. Compositional Analysis

The composition of the carbon nanofluids was changed during the experiments as Table S1.

Table. S1. Compositional change of graphite and CNT in the experiment.

Sample	Initial concentration (wt%)		Final concentration (wt%)	
	Graphite	CNT	Graphite	CNT
1	1	0	0.203	0
2	0.75	0.25	0.151	0.056
3	0.5	0.5	0.097	0.106
4	0.25	0.75	0.018	0.141
5	0	1	0	0.156

2. Structural analysis

The XRD results was presented in Figure S1.

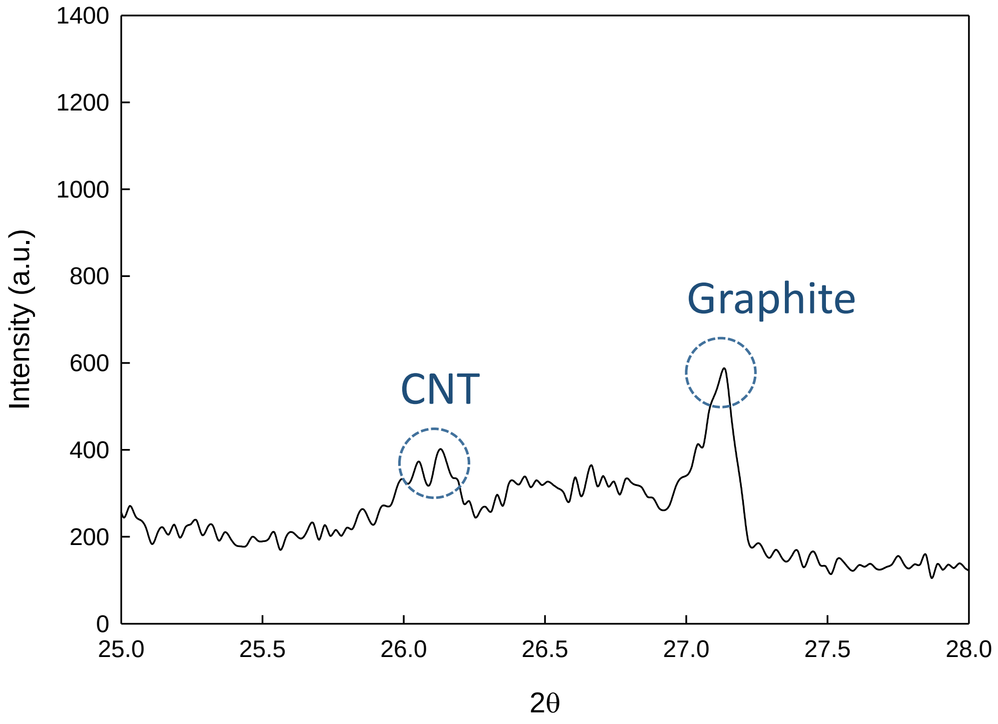

Fig. S1. XRD graph of carbon nanomaterials.

3. Size analysis

The size of sample 1 was analyzed as shown in Figure S2.

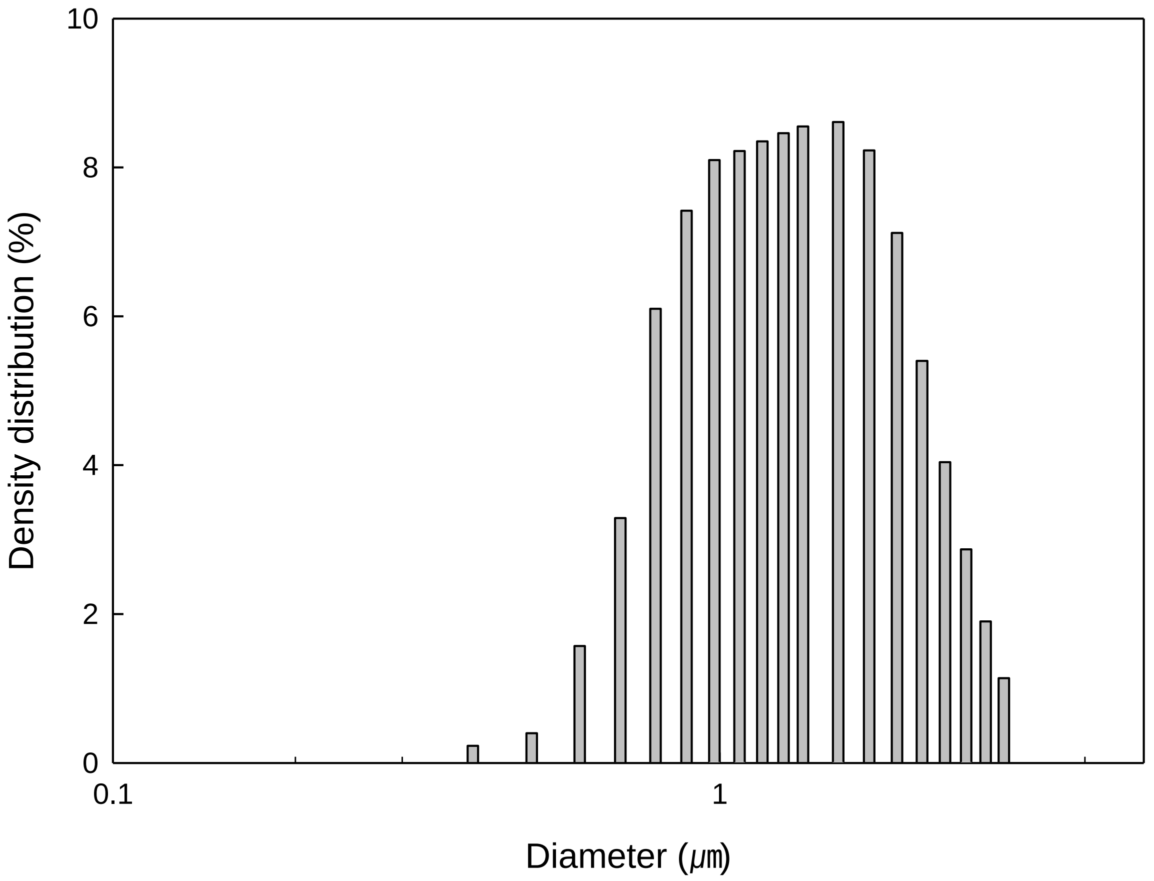

Fig. S2. Size distribution of sample 1.

4. Thermal conductivity

The thermal conductivity of carbon nanofluids were evaluated using the Hamilton-Crosser model as follows:

$$\frac{k_e}{k_f} = 1 + \frac{n(\alpha - 1)\varphi}{(\alpha + n - 1) - (\alpha - 1)\varphi}$$

where k_e and k_f are the effective thermal conductivities of the suspension and the base fluid, respectively, $\alpha = \frac{k_p}{k_f}$ is the k ratio, k_p is the particle conductivity, n is the particle shape factor, and φ is the particle volume fraction.

To measure the thermal conductivity of the carbon nanofluids, we employed a modified transient plane source technique (MTPS), which uses a one-sided, interfacial, and heat reflectance sensor. This method is capable of measuring thermal conductivities of various matters such as liquids, powders, pastes, and solids. The tests were carried out five times for each sample, and the resulting standard deviations of the tests were 0.061 (sample 1), 0.057 (sample 2), 0.062 (sample 3), 0.059 (sample 4), and 0.058 (sample 5).

5. Rheological analysis

The power law index and flow consistency index were presented in Table S2.

Table S2. Power law index and flow consistency index of samples.

Sample	Power law index (*n*)	Flow consistency index (*K*)
Sample 1	0.9685	0.001352
Sample 2	0.956	0.001588
Sample 3	0.0466	1.11815
Sample 4	-0.2207	37.17064
Sample 5	-0.3052	82.20533

6. Morphological analysis

The carbon nanofluids were air-dried, and their morphologies were analyzed with a field emission scanning electron microscopy as shown in Figure S3.

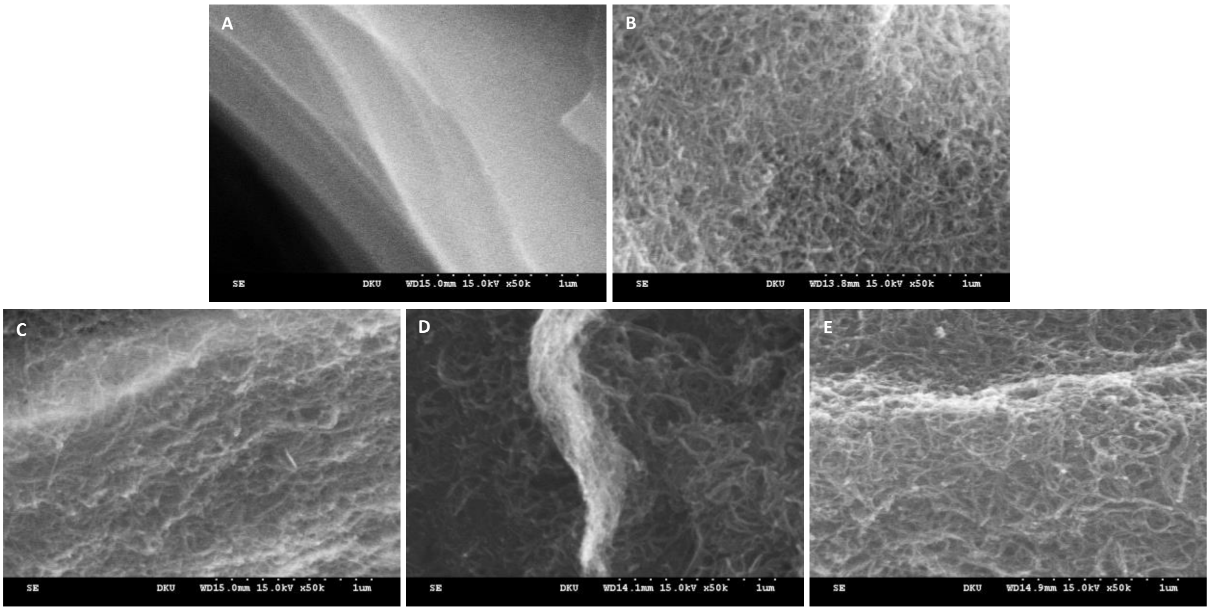

Fig. S3. FESEM images of (a) sample 1, (b) sample 2, (c) sample 3, (d) sample 4, and (e) sample 5.

7. Electrochemical analysis

The results of electrochemical analysis were shown in Fig. S4 and S5.

A

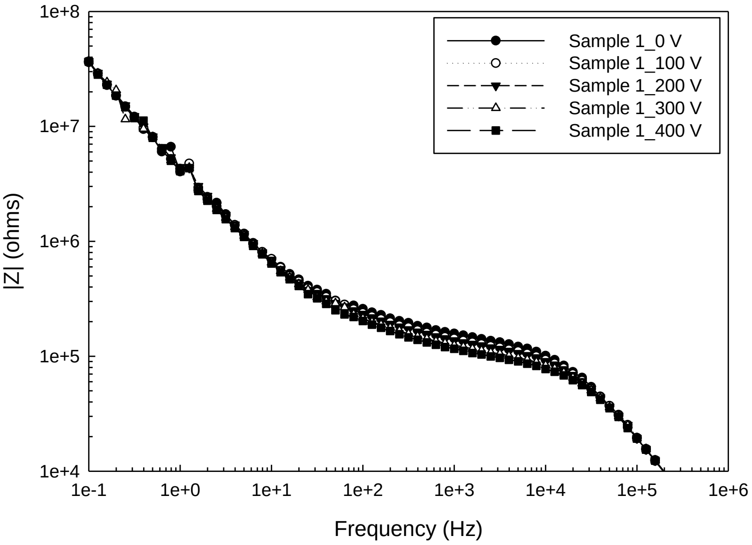

B

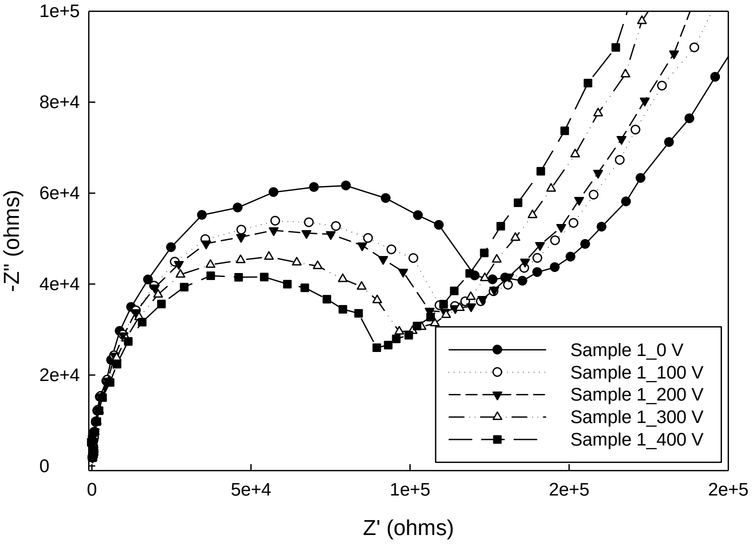

Fig. S4. Microfluidic impedance changes according to the applied potentials: (a) Bode plot of sample 1, (b) Nyquist plot of sample 1.

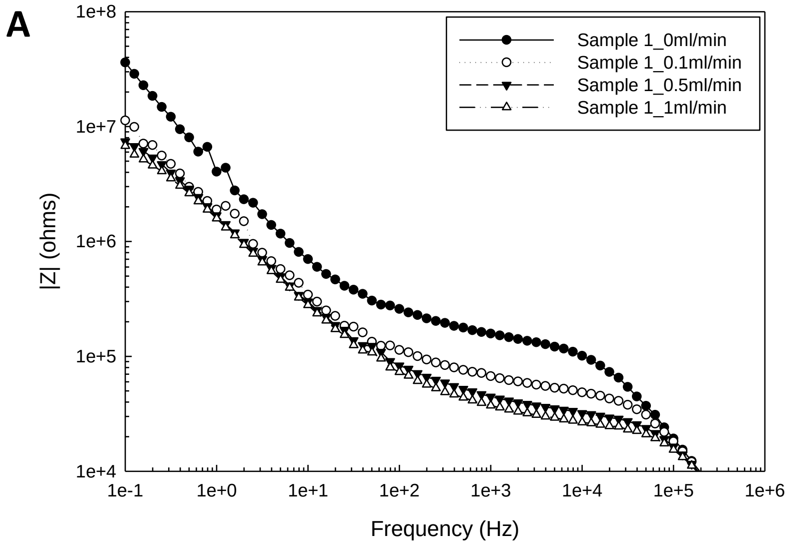

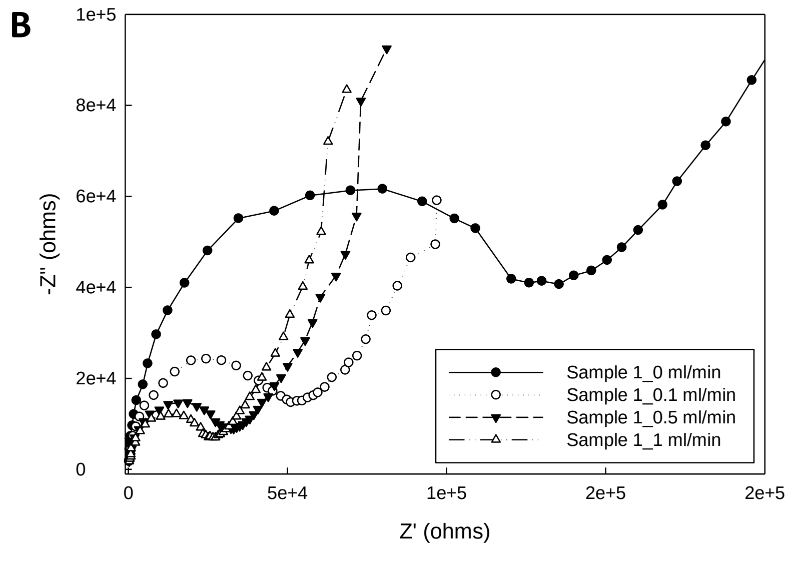

Fig. S5. Microfluidic impedance changes according to the applied flow fields: (a) Bode plot of sample 1, (b) Nyquist plot of sample 1.
